# The Stop-Tabac smartphone application for smoking cessation: study protocol for a randomized controlled trial in the general population

**DOI:** 10.1186/s13063-020-04377-0

**Published:** 2020-06-01

**Authors:** Jean-François Etter, Yasser Khazaal

**Affiliations:** 1 Institute of Global Health, Faculty of Medicine, University of Geneva, 9 chemin des Mines, Campus Biotech, CH-1202 Geneva, Switzerland; 2 Addiction Medicine, Department of Psychiatry, Lausanne University Hospital and Lausanne University, Lausanne, Switzerland

**Keywords:** Smoking, Smoking cessation, Randomized controlled trial, Tobacco dependence, Nicotine replacement therapy, Smartphone, Mobile phone

## Abstract

**Background:**

Smartphone-based support can reach thousands of smokers and help those who would otherwise try to quit smoking by themselves with little chance of success. Nicotine medications double the chances of quitting smoking, but few smokers use them, and they often use them for too short a time and at an insufficient dose. It is therefore important to increase access to support for smoking cessation and compliance with nicotine therapy. The objectives of this study are to assess whether the Stop-Tabac application (app) is effective for smoking cessation and to examine whether the outcome is influenced by the personal characteristics of participants.

**Methods:**

Trial design: this is a two-arm, parallel-group, superiority, individually randomized, “placebo” controlled trial in 5200 smokers, with follow up after 1 week, 1 month and 6 months. The participants are adult daily smokers (*N* = 5200) enrolled on the Internet, living in France or Switzerland. The intervention is the Stop-tabac fully-automated app for smartphones, which was launched in 2012 and continuously improved thereafter. It includes fact sheets; calculators of cigarettes not smoked, money saved, and years of life gained; an interactive “coach” that provides automated, individually tailored counseling messages based on the user’s personal profile, sent regularly for 6 months; immediate feedback during episodes of craving and tobacco withdrawal symptoms; a discussion forum (“The Tribe”) where participants provide and receive social support; a quiz that informs users in a playful way; and a module on nicotine therapy that includes personalized feedback and follow up. The outcome is self-reported smoking cessation after 6 months (no puff of tobacco in the past 4 weeks), and after 1 week and 1 month (no puff in the past 7 days). Participants will be randomized automatically based on a list of random numbers. Participants, assistants in charge of collecting follow-up data and data analysts will be blinded to allocation. Funding is provided by the Swiss National Science Foundation, CHF 194,942 (EUR 182,200, USD 200,700), grant 32003_179369. JFE’s salary is paid by the University of Geneva, YK’s salary is paid by the Lausanne University Hospitals.

**Discussion:**

There is little evidence from randomized trials of the impact of health apps in general and of smoking cessation apps in particular. This study will fill this gap.

**Trial registration:**

ISRCTN Registry: ISRCTN11318024. Registered on 17 May 2018.

## Background

### Current state of research in the field

Tobacco smoking is the first avoidable cause of death and disease in developed countries [1, 2]. The mass level dissemination of smoking cessation interventions remains one of the most effective and cost-effective ways to decrease smoking prevalence and subsequently mortality from cancer, cardiovascular diseases and lung diseases [3, 4].

When smokers try to quit, 70% of them try by themselves without professional help or medications [5]. Among people who try to quit smoking by themselves, smoking abstinence rates after 12 months are as low as 3–5% [6]. Smokers rarely seek professional support, because of time constraints or the financial cost, or because many healthcare providers do not offer satisfactory smoking cessation support [7]. Few smokers use pharmacotherapy during quit attempts, either because of misconceptions about the nature of addiction and about the benefits and risks of these medications or because of their cost [8]. Many smokers overestimate the risks of nicotine medications, and observance is poor among users [9, 10]. Therefore, increasing the proportion of smokers who try to quit, improving the success rate in those who try, and increasing the use of behavioral support and of nicotine therapy during quit attempts are public health priorities. It is necessary to extend smoking cessation counseling beyond specialized clinics, doctors’ offices, and telephone helplines, in order to reach the thousands of smokers who never receive support from these sources. Mobile phone-based interventions are an optimal tool for this, because they are available 24 h a day/7 days a week/365 days a year, everywhere and often at no charge. A review concluded that, at a global level, text messaging on mobile phones is one of the most affordable effective interventions to assist tobacco cessation [11]. A Cochrane meta-analysis showed that old-fashioned text messaging on mobile phones is effective for smoking cessation (relative risk 1.67), although several of the included studies showed no effect [12]. The authors of this review did not find any randomized trial of modern smartphone apps that fulfilled their inclusion criteria and recommend that more trials should be conducted in this field [12].

#### Smartphone-based smoking cessation support

There is currently a multi-billion-dollar industry in smartphone applications (apps) that disseminate health information on many topics and provide individually tailored support at population level [13–15]. Potentially, smartphone apps could be more effective than text messaging, because the best apps include several features in addition to text messaging (e.g. discussion forums and individually tailored feedback). However, literature reviews conclude that the average quality of apps for smoking cessation is poor, that few apps adhere to guidelines for treating tobacco dependence, that few apps provide individually tailored feedback, and that most apps use only simplistic tools (e.g. calculators of cigarettes not smoked and money saved) [13, 16–18]. One review concludes that in the field of smoking cessation, only 4% of the 50 most commonly downloaded apps have any scientific support, and that only half of the scientifically vetted apps were still available to consumers at the time of the review [17].

There are few published randomized controlled trials (RCT) of smartphone apps for smoking cessation; these trials were conducted in small samples, their results are inconclusive [19–23], and a recent review concludes that good quality trials in large samples are needed [24]. We know of no published randomized trial in a sample large enough to detect a sustained effect of these apps on smoking cessation. The present study will fill this gap in the literature: it includes an adequate control group, sufficiently long follow up (6 months) to fulfill the inclusion criteria in the Cochrane reviews, and it includes a sample large enough to detect a statistically and clinically significant effect.

There is a need for high quality, professionally developed, effective smartphone-based smoking cessation interventions. The Stop-tabac application was developed to fulfill these criteria: its authors are experienced specialists in the field of tobacco dependence, the app is based on theory and on the scientific evidence, and it provides individually tailored advice. The app was iteratively improved over the past 8 years based on feedback received from users [25]. A recent academic review ranked the Stop-Tabac app among the best five smoking cessation apps worldwide [18].

#### Nicotine replacement therapy

Nicotine replacement therapy (NRT) multiplies the chances of quitting smoking by 1.6 compared with placebo [26], and clinical guidelines recommend NRT as a first line treatment for tobacco dependence [27, 28]. However, too few smokers use NRT during quit attempts [5, 29]. Many smokers who use NRT do so without behavioral support, which means that they do not obtain the full benefit this treatment can offer, in particular because of poor compliance: they use too small doses during too short a time [9, 10, 30, 31]. Smokers also have misconceptions about the efficacy and risks of NRT [10]. To address this problem, the Stop-tabac app includes a module aimed at increasing the use of and compliance with NRT.

#### Combining nicotine therapy with smartphone-based behavioral support

Research shows that compliance with NRT increases with the amount of behavioral support received [26], and that behavioral support increases quit rates in people using NRT [32]. However, few NRT users have access to behavioral support. Therefore, there is a need to produce mass-level behavioral support for people who either are prescribed NRT by their doctor but do not receive behavioral support, or who purchase NRT without a medical prescription. Adding smartphone-based support to nicotine therapy represents an opportunity to increase the effectiveness of both components, including by improving compliance with NRT (better dosage and longer treatment duration). Several studies showed that interactive, computer-based behavioral treatments combined with NRT can improve quit rates better than NRT alone [33–35]. Whether smartphone apps can produce the same effect has not been explored.

### In summary, what is not known on this topic

Even though over 400 different smoking cessation apps are available, the quality of most of them is poor [13, 16–18], there are few published randomized trials of smartphone apps for smoking cessation, most of these trials were conducted in very small samples, and their results are inconclusive overall [19, 20, 22, 24]. It is not known whether these apps actually help people stop smoking and maintain abstinence over several months, neither is it known whether these apps elicit quit attempts in current smokers or improve NRT usage. Our study will address these important questions, using a sample size much larger than in previous trials, which will provide sufficient statistical power. Our study includes a control group that uses a “placebo” app, which reinforces the likelihood that any observed association will be causal.

The value of the mobile health industry is projected to be 59 billion USD in 2020 [15]. Health apps are massively used, even though very few of them have ben rigorously evaluated, it is therefore important to evaluate these apps in randomized controlled trials.

## Methods/design

### Objectives

The objective is to test whether the Stop-tabac smartphone application (iOS and Android) improves smoking cessation rates in current smokers and helps them maintain abstinence over 6 months, compared with quit rates observed in a control group, and to measure the size of this effect.

Secondary objectives are:

1. To study whether the outcome is influenced by the characteristics of participants, such as dependence level, demographics (sex and age), smoking history, and depression.
2. To assess whether the app has any effect on motivation to quit, quit attempts, use of NRT, e-cigarettes and heated tobacco products, number of cigarettes smoked per day, and perceived usefulness of the app.

### (Fig. 1 ) Study design

This is a two-arm, parallel-group, superiority, individually randomized, “placebo” controlled trial with follow up after 1 week, 1 month and 6 months.

**Fig. 1:**
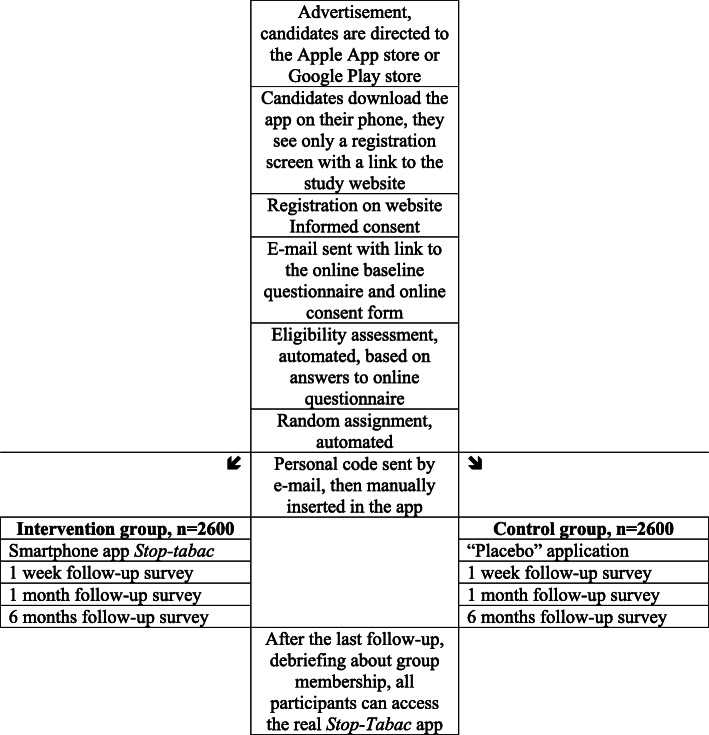
Flowchart of participants

#### Participants and recruitment

Participants are 5200 smokers who live in Switzerland and in France. The study is advertised on the Internet and social networks, and by smoking prevention organizations. Recruitment is through self-identification and self-selection.

#### Outcomes

##### Primary outcome

The primary outcome is smoking cessation: the proportion of participants at the 6-month follow up who report they have not smoked even a puff of tobacco in the previous 4 weeks.

##### Secondary outcomes

Secondary outcomes are:

- Smoking cessation: proportion of participants who report they have not smoked even a puff of tobacco in the previous 7 days, assessed at each follow-up.
- Smoking cessation as defined in the Russell Standard [36]: proportion of participants at the 6-month follow up who report they smoked ≤5 cigarettes in the past 6 months but did not smoke even a puff of tobacco in the past 7 days, and proportion of participants at the 4-week follow up who report they smoked ≤ 5 cigarettes in the past 4 weeks but did not smoke even a puff of tobacco in the past 7 days.
- Abstinence from both smoking and vaping: the proportion of participants who report they did not smoke any tobacco or use nicotine-containing e-cigarettes or heated tobacco products after their target quit date, assessed at each follow up.
- Nicotine use: the proportion of participants who used nicotine therapy, nicotine-containing e-cigarettes or heated tobacco after their target quit date, assessed at each follow up.
- Perceived usefulness of the app: the question reads: “Did the *Stop-Tabac* app for mobile phones help you quit smoking?”, and is answered on a Likert-type scale with 6 response options ranging from “Yes, enormously” to “On the contrary, it made me smoke or start smoking again”, assessed at each follow up.
- App use: the proportion of participants who report using any smoking cessation app after their target quit date, assessed at each follow up.

Among those who smoke at follow up:

- Intention to quit smoking: the proportion of participants who answer each of the response options to the question: “Do you intend to quit smoking? (response options: I have firmly decided to quit/I consider quitting/No), assessed at each follow up.
- Quit attempts: the proportion of participants who report they seriously tried to quit smoking after their target quit date (response options: yes/no), assessed at each follow up.
- Amount smoked: mean number of cigarettes smoked per day, and change since baseline in cigarettes per day (mean change score), assessed at each follow up.

Secondary outcomes from data collected automatically by the app:

- Duration of app use (mean number of days between the first and last use of the app) and frequency of app utilization (mean number of times the app was opened), recorded automatically at individual level, assessed at 6-month follow up only.

Inclusion criteria:

1. Daily cigarette smoker
2. Has been a daily smoker for at least 1 year
3. Age ≥ 18 years
4. Sets a target quit date within 1 month of enrollment, and commits to quit on this date
5. Provides informed consent online
6. Commits to complete all follow-up questionnaires
7. Commits to use the app
8. Owns a smartphone (Android or iOS) and has regular access to e-mail
9. Provides a postal address, a telephone number, and a valid e-mail address
10. Lives in Switzerland or in France

The exclusion criterion is prior use of the Stop-tabac app for smartphones (self-report).

#### Informed consent

The online consent form describes the study, risks and benefits, and how confidentiality is maintained, and explains the data collection and data sharing procedures. We will wait until the end of the study to inform participants of the control group procedure and of the placebo version of the app. Participants receive the intervention at no charge and are not paid.

#### Enrollment

Interested participants are directed to the Apple App Store and Google Play Store, where the app will remain available during the whole study and where they can download the app on their smartphone. At this point, the app only displays a screen that instructs participants to visit the study website where they can obtain a personal code that gives them access to the app. Once on the website, participants read an information page about the study, complete a consent form, and indicate their e-mail address. Participants access the baseline screening questionnaire through a link in an e-mail message, which ensures that we have a valid e-mail address for each of them.

#### Randomization

Participants are randomized only after we receive the baseline survey and the consent form, and after we verify eligibility. Eligibility is assessed automatically by computer algorithms. We perform an automated check of e-mail addresses, names, age, and sex, to avoid double registration of participants. Once a candidate is declared eligible, randomization is automatically performed by a computer using a list of random numbers, with a 1:1 ratio and using simple randomization, with no strata or block. After randomization, participants receive a personal code number in an e-mail message, they insert this code number manually in the app on their smartphone, and at this point they can access either the complete Stop-tabac app or to the placebo app. This procedure ensures that participants register only once, that all of them open the app, and that they have access to their intended intervention only. Participants who do not manually enter their personal code in the app do not have access to any version of the app and are excluded after randomization. Access to the app is restricted to study participants during the study; non-participants cannot access or download the app.

#### Blinding

Participants are blinded to their assignment group and to the existence of a placebo group. The placebo app is also named Stop-tabac - it looks almost the same as the complete app but with fewer features. Thus, control participants may not easily realize that they receive a placebo app. Participants will be informed about the placebo-controlled study design after the end of the study only, and they will be given access to the full version of the app at this point. It is ethical to avoid informing participants of the placebo procedure, because informing them could jeopardize the blinding and stimulate them to use other smartphone apps, thus contaminating the control group. It is ethical to use a placebo-controlled study design because we do not know whether the Stop-tabac app or any other similar app is effective for smoking cessation.

For baseline and follow-up surveys, online data collection is fully automatic, and thus, there is no bias in online assessments. People who do not respond to the online survey receive the follow-up questionnaires by postal mail, and those who do not respond to the postal communication answer the questionnaire over the phone. Assistants who send text or WhatsApp reminder messages and who collect questionnaires by post and over the phone are blinded to the group assignment of participants, to avoid bias. The data analyst is blinded too. The only person with access to the code linking names and group membership is a computer expert who works under the supervision of the first author (JFE), and the data will be unblinded only after the main analysis is finished. There will be no unblinding during the trial.

#### Test of procedures

To ensure that the trial runs smoothly, all procedures (data collection, reminders, supervision of participants) were tested in 15 people (7–8 in each study group) before the study started. In this test, procedures were tested until the 1-month follow-up survey only (i.e. not at 6 months).

### (Fig. 2) Intervention and control group procedures

#### Procedures for both study groups

Registration in the study is accessible only to participants who download the app; this ensures that all participants install the app on their phone. We do not ask participants to avoid using other smoking cessation treatments during the study because this would be unethical.

**Fig. 2:**
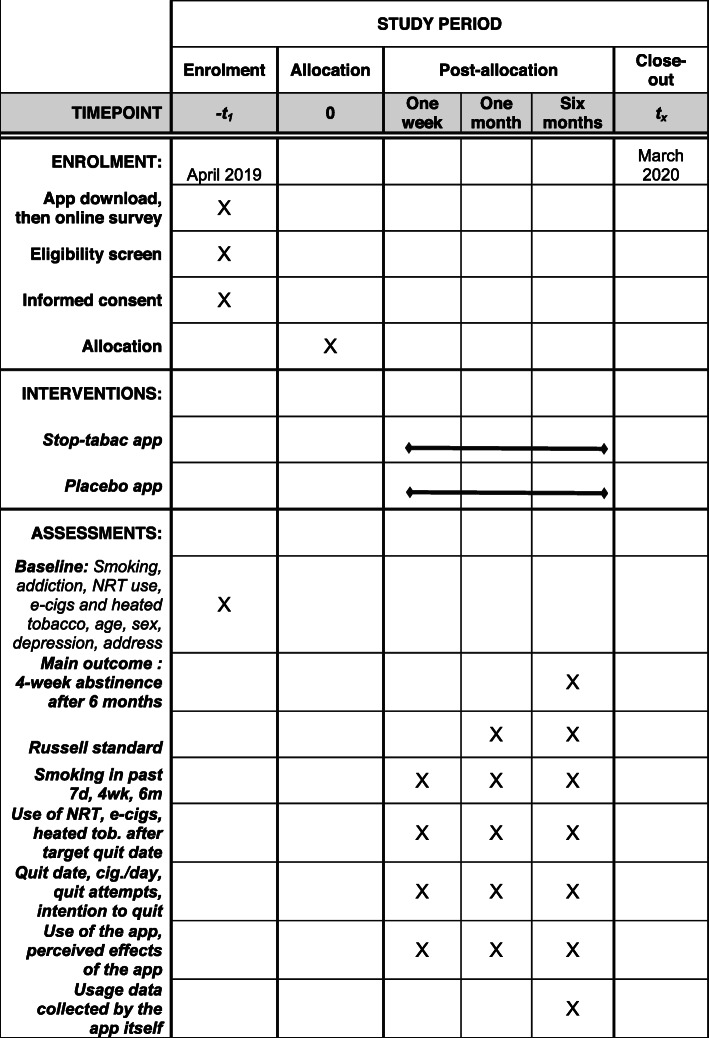
Schedule of enrolment, interventions, and assessments. NRT, nicotine replacement therapy

#### Intervention: the Stop-tabac application for smartphones

The Stop-tabac app is available for iOS (Apple) and for Android, and it was used by 24,000 people every month before we started this study. We used two strategies to ensure that prior users of the app did not register in the trial, and to enroll only new, naïve users. First, we deactivated new downloads one month before we started the study. Because users usually use the app for only a few days or weeks (very few use it for more than 4 weeks), this strategy gradually purged the stock of current users before the study started. Second, in the baseline survey, we asked participants whether they had already used the Stop-Tabac app and we excluded those who had. These two strategies ensure that the study results are not influenced by participation in the control group of prior users of the app.

This smartphone app, in French, is a stand-alone intervention intended at motivating and helping smokers quit smoking. It has been developed since 2012 by experts in addiction and continuously improved thereafter, taking into account the suggestions made by users in face-to-face tests and in online satisfaction surveys. The app is based on behavior change and addiction theories [37–39], on guidelines and literature reviews on treatments for tobacco dependence [11, 27], and on the applicants’ research [40] and experience with smokers. The app includes:

- Brief texts on tobacco dependence, tobacco withdrawal symptoms, tobacco dependence treatments, relapse situations, mood management, risks of smoking and benefits of quitting, e-cigarettes, and heated tobacco products.
- Calculators (number of cigarettes not smoked, money saved since quitting, additional days of life expectancy gained since quitting).
- Immediate feedback when participants experience challenging situations (e.g. relapse situations, craving episodes).
- A quiz (42 questions), which allows the transmission of knowledge in a playful way.
- A link that automatically dials the phone number of the national quit-lines in France or Switzerland.
- The “Coach”, an automatic system that produces individually tailored feedback messages and sends periodic reminders (push notifications within the app). Each participant receives individually tailored advice and follow-up messages based on their target quit date, level of tobacco dependence, perceived advantages and drawbacks of smoking, and motivation to quit. The duration of this automated intervention is 6 months. Participants can disable the push notifications.
- A discussion forum, “The Tribe”, permanently (24 h a day/7 days a week/365 days a year) moderated by a psychologist helped by volunteers under her supervision. An influential guideline identified social support as an important and effective component of smoking cessation interventions [27]. The “Tribe” is quite active and is very appreciated by users.
- A module on nicotine replacement therapy (NRT) that includes fact sheets (NRT utilization, benefits and side effects, where and how users can obtain NRT); frequently asked questions (FAQ); and a series of brief individually tailored feedback messages, based on users’ responses to questions on current NRT use, knowledge about NRT, intention to use NRT, craving and withdrawal symptoms. If users report withdrawal symptoms and if they have quit smoking for < 3 months, they receive automated messages telling them to use NRT. These messages insist on the importance of using a sufficient dose of nicotine and not interrupting the treatment prematurely.
- A module on electronic cigarettes that includes a series of brief individually tailored feedback messages, based on users’ responses to questions on current vaping, intention to use e-cigarettes, opinions about the effects of vaping on smoking cessation and nicotine withdrawal symptoms, perceived side-effects, and perceived addictiveness of e-cigarettes.

The Stop-tabac.ch application belongs to the University of Geneva, it is available at no charge on the app stores, and the investigators of this study and the authors of the app have no financial interest in this app, and no conflict of interest with the pharmaceutical, tobacco or e-cigarette industries. The development and maintenance of the Stop-tabac app are supported by the Swiss Tobacco Prevention Fund (at the Swiss Ministry of Health), and the app does not receive support from the pharmaceutical, tobacco, or e-cigarette industries. During the study, the app is only available to study participants.

The conditions of use of the app in the trial are similar to the conditions of use outside the trial. In particular, the frequency of use of the app is determined by the users themselves, and we do not use any prompts or reminders to trigger utilization of the app (no SMS or e-mail), apart from the notifications normally sent within the app itself.

#### Control group procedures

The placebo app includes a few features that are liked by users (brief information pages, calculators of money saved, cigarettes not smoked, and days of life expectancy gained). We do not think that this content is sufficient to produce an impact, but it should prevent the potential problem that the placebo app is so basic and unsatisfactory that control participants access other support more frequently than intervention participants, which may bias the results in the direction of null findings. After randomization, we only contact participants in both study groups for the three follow-up surveys.

#### Study software and data protection

A special software was set up to manage the study, enroll participants, and conduct baseline and follow-up surveys. Participants’ information is kept confidential and accessible only to the members of the research team (the project leaders, a computer expert and two research assistants). The dataset for analysis will be anonymized. The only person who can access the code linking participants’ names and their responses to questionnaires is a computer expert under the supervision of the first author.

#### Data to be collected

The primary outcome is self-reported smoking abstinence after 6 months (no puff of tobacco in the past 4 weeks). This is the criterion recommended by the American Food and Drug Administration (FDA) to assess outcome in smoking cessation studies [41]. We also assess outcome using the “Russell Standard”, a recently suggested standard for smoking cessation trials: continuous 6-month (at 6 months) or 1-month (at 1 month) abstinence allowing for smoking ≤ 5 cigarettes after the target quit date, but no cigarette in the past 7 days [36].

#### Baseline data

The baseline online questionnaire, in French, is administered through a mobile-friendly website, accessible via any platform: smartphone, tablet, or computer. Baseline variables include smoking behavior, tobacco dependence, current use of nicotine medications, e-cigarettes, and heated tobacco (Iqos, Glo, Ploom, or other brand), age and sex, a brief (two items) screening test for depression [42], postal address, telephone number, and e-mail address.

#### Follow up after 1 week, 1 month and 6 months

The date for follow-up surveys is tied to the target quit date set at baseline. Follow-up data are collected online, via a questionnaire accessible on all platforms. Participants receive an invitation by e-mail to answer the follow-up questionnaires 1 week, 1 month and 6 months after their target quit date. After reminders via e-mail (7 days: four reminders; 1 month: six reminders; 6 months: eight reminders), this online data collection is fully automatic. The next steps only involve a human intervention: assistants send non-responders three reminders via text messages (or WhatsApp) on their cellphones, then non-respondents receive the follow-up questionnaire by post, and those who do not respond to the postal questionnaire are contacted by phone [43]. Follow-up questionnaires are brief and focused, in order to maximize participation.

#### No biochemical verification of smoking cessation

The Society for Research on Nicotine and Tobacco (SRNT) guidelines for assessing outcome in smoking cessation studies do not recommend biochemical verification when there is no close contact or interaction between participants and the study team [44]. Although the absence of biochemical verification may result in some under-reporting of smoking, this should not differ between the intervention and control groups, so this should not impact the test of effectiveness.

#### Potential bias

Participants consist of volunteers and may not be representative of all smokers. We will compare our sample to representative samples of smokers in Switzerland and France. There are no restrictions to obtaining smoking cessation support outside the study. We ask participants whether they used NRT, e-cigarettes, heated tobacco, or smoking cessation apps during the study, and will use this information in our analysis and interpretation of the results.

There is a risk that participants in the more engaging “complete” app (with push notifications) will be more burdened and delete the app or disable the app notifications, but the current content and frequency of reminders have been considered acceptable by app users over the years.

All answers are given via drop-down menus or radio buttons (except names and addresses), and thus only valid answers are recorded. At baseline, participants are required to answer all questions (an incomplete form cannot be sent) and thus there are no missing data. At follow up, answers to the questions on smoking status (any smoking in the past 7 days, 4 weeks, and 6 months) are required, but participants can send an incomplete form as long as they answer these questions. An assistant will enter the data for questionnaires on paper (collected by post or over the phone) by single data entry.

#### Sample size calculation

Based on the literature on Internet-based text messaging and smartphone interventions [12, 45], and on our own prior studies of Internet-based interventions [40, 46], we expect quit rates of 12.5% in the complete app group and 10% in the placebo group after 6 months (odds ratio = 1.28). The difference of 2.5 percentage points may appear to be small, but it is about half of the effect of nicotine replacement therapy combined with medical counseling (6% above placebo) [26], or the effect of bupropion (5% above placebo) [47], or the effect of intensive smoking cessation counseling by physicians (5% above usual care) [48]. Thus, an effect of 2.5 percentage points is actually quite large, and it would not be reasonable to expect a much larger effect. An effect of 2.5 percentage points applied to tens of thousands of users translates into a substantial impact at population level. A total of 5200 smokers (2 × 2600) will enable us to detect this effect with power of 80% and a confidence level of 95%.

#### Intention to treat, missing data

For the primary outcome, participants will be evaluated in an intention-to-treat analysis, with the baseline number of participants as the denominator. Participants absent at follow-up will be counted as smokers, except those who are dead. We will conduct sensitivity analyses with different assumptions for missing data. For each follow-up questionnaire, non-responders will be counted as smokers for this specific questionnaire. A participant who is missing at the 1-month follow up but then returns as a non-smoker for the 6-month follow up will be counted as smoker for the 1-month follow up only.

#### Discontinuation criteria, protocol modifications

Participants can withdraw from the study at any point, without having to justify their decision. Participants need to inform the researchers to be classified as withdrawing. Participants who withdraw from the study are not given access to the full app, but the full app will be freely accessible to all after the end of data collection.

There is no plan to amend or modify the study protocol. If the protocol must nevertheless be modified, we will amend the ISRCTN record, and we will mention in all published papers that the protocol was modified during the study, and how it was modified. Assistants who contact non-respondents worked at home during the lockdown during the epidemic in Spring 2020, performing their usual tasks.

#### Risks to participants

There is no risk associated with the behavioral program and the data collection procedure. There is a risk that data may be accessed by hackers. We make every effort to minimize this risk by using appropriate security measures.

#### Relationships with the industry

The investigators of this study and the authors of the smartphone app are independent from all commercial interests, in particular from the pharmaceutical, tobacco, and e-cigarette industries.

#### Dissemination policy

Results will be published in peer-reviewed journals and presented at conferences; there is no publication restriction. Both co-investigators (JFE and YK) will be co-authors; if there are any other contributors, authorship will be granted according to the usual rules. We do not intend to use professional writers. After publication of the study results, participant-level data and the statistical code will be made publicly accessible in an anonymous format.

#### Confidentiality

The data are stored on computers at the University of Geneva and at an external provider, with high levels of security. The dataset for analysis will be anonymized. The only person who can access the code linking participants’ names and their responses to questionnaires is a computer expert under the supervision of the first author. Data will be anonymized before they are made publicly available on a repository.

### Statistical analyses and data management

The main analysis will be a comparison of the proportions of abstinent smokers (as defined above) after 6 months in the intervention and control groups. We will use the chi-square test and odds ratios with 95% confidence intervals to compare proportions. We will also conduct subgroup analyses and will use multivariate models to test whether the outcome is associated with participants’ characteristics and with smoking cessation tools obtained outside the study. We will assess whether the outcome is associated with the frequency of utilization of the app (from usage data automatically collected by the app). The study is conducted and results will be presented in conformity with the Consolidated Standards of Reporting Trials (CONSORT) guidelines [49] and CONSORT E-Health checklist [50]. Creating a data monitoring committee was not deemed necessary because the data are collected mostly automatically, and because only valid answers can be entered in the online forms via drop-down menus or radio buttons, thus no major quality issues arise with this data set. The data will be accessible to the two co-investigators (JFE and YK), and after publication of the study results, participant-level data and the statistical code will be made publicly accessible in an anonymous format.

## Discussion

### Scientific relevance

A randomized trial comparing a real application with a placebo application is an innovative approach. The placebo-controlled design will increase our ability to derive causal inferences from the data. This study will help us understand how smartphones can be used to promote smoking cessation and to improve use of pharmacological treatments. The study will also provide information on the categories of smokers in whom this intervention is most effective.

### Broader impact

In the last few years, a multi-billion dollars industry of health-related smartphone applications has appeared [15], but there is little scientific evidence from randomized trials of the impact of these apps in general, and of smoking cessation apps in particular [12]. The Stop-Tabac app is free of charge, it is ranked among the best smoking cessation apps worldwide [18], and it is already widely disseminated (24,000 monthly users before we restricted access to study participants only). If this study proves that this app is effective, this would be a strong argument in favor of directing more resources to smartphone-based interventions, and this approach could be widely disseminated and save many lives. Thus, this innovative study has major implications for smoking prevention policy and for mobile health in general.

## Trial status

Protocol version 1, 25 February 2020. The recruitment of participants started in May 2019 and was completed in March 2020.

## Data Availability

A data management plan was submitted as required by the Swiss National Science Foundation. After publication of the main results, the anonymized data will be made publicly available. The shared SPSS data file, with participant-level data, will be fully documented, which means that the variable labels and value labels will include the full wording of questions and of response options. An accompanying Readme.txt file will include a full description of data collection procedures and analyses.

## Abbreviations

app: Smartphone application
NRT: Nicotine replacement therapy
